# Supplementary material for: How Effective Are Push–Pull Semiochemicals as Deterrents for Bark Beetles? A Global Meta-Analysis of Thirty Years of Research
Source: Insects. 2023 Oct 13;14(10):812. doi: 10.3390/insects14100812 (PMC10607257; doi:10.3390/insects14100812)
Supplement: Supplementary file 1 [file insects-14-00812-s001.zip › S1_Sup_selected_pappers.pdf]

1. ANDERSSON, M. N., BINYAMEEN, M., SADEK, M. M. & SCHLYTER, F. 2011. Attraction Modulated by Spacing of Pheromone Components and Anti-attractants in a Bark Beetle and a Moth. Journal of Chemical Ecology, 37, 899-911.
2. ANDERSSON, M. N., LARSSON, M. C., BLAŽENEC, M., JAKUŠ, R., ZHANG, Q. H. & SCHLYTER, F. 2010. Peripheral modulation of pheromone response by inhibitory host compound in a beetle. Journal of Experimental Biology, 213, 3332-3339.
3. AUDLEY, J. P., FETTIG, C. J., MUNSON, A. S., BLACKFORD, D. C., MORTENSON, L. A. & MAFRA-NETO, A. 2022. MCH-Based Semiochemical Repellents for Protecting Engelmann Spruce Trees from *Dendroctonus rufipennis* (Coleoptera: Curculionidae). Journal of Economic Entomology, 115, 187-192.
4. BENTZ, B. J., KEGLEY, S., GIBSON, K. & THIER, R. 2005. A Test of High-Dose Verbenone for Stand-Level Protection of Lodgepole and Whitebark Pine from Mountain Pine Beetle (Coleoptera: Curculionidae: Scolytinae) Attacks. Journal of Economic Entomology, 98, 1614-1621.
5. BYERS, J. A. 1993. Avoidance of competition by spruce bark beetles,*Ips typographus* andPityogenes chalcographus. Experientia, 49, 272-275.
6. CAMPBELL, S. A. & BORDEN, J. H. 2006. Integration of visual and olfactory cues of hosts and non-hosts by three bark beetles (Coleoptera: Scolytidae). Ecological Entomology, 31, 437-449.
7. DODDS, K. J. & MILLER, D. R. 2010. Test of nonhost angiosperm volatiles and verbenone to protect trap trees for *Sirex noctilio* (Hymenoptera: Siricidae) from attacks by bark beetles (Coleoptera: Scolytidae) in the northeastern United States. Journal of Economic Entomology, 103, 2094-2099.
8. ETXEBESTE, I. & PAJARES, J. 2011. Verbenone protects pine trees from colonization by the six-toothed pine bark beetle, *Ips sexdentatus* Boern.(Col.: Scolytinae). Journal of Applied Entomology, 135, 258-268.
9. FETTIG, C. J., BORYS, R. R., DABNEY, C. P., MCKELVEY, S. R., CLUCK, D. R. & SMITH, S. L. 2005a. Disruption of red turpentine beetle attraction to baited traps by the addition of California fivespined ips pheromone components. The Canadian Entomologist, 137, 748-752.
10. FETTIG, C. J., MCKELVEY, S. R., BORYS, R. R., DABNEY, C. P., HAMUD, S. M., NELSON, L. J. & SEYBOLD, S. J. 2009. Efficacy of verbenone for protecting ponderosa pine stands from western pine beetle (Coleoptera: Curculionidae: Scolytinae) attack in California. Journal of Economic Entomology, 102, 1846-1858.
11. FETTIG, C. J., MCKELVEY, S. R., DABNEY, C. P. & HUBER, D. P. W. 2012. Responses of *Dendroctonus brevicomis* (Coleoptera: Curculionidae) in behavioral assays: Implications to development of a semiochemical-based tool for tree protection. Journal of Economic Entomology, 105, 149-160.
12. FETTIG, C. J., MCKELVEY, S. R. & HUBER, D. P. W. 2005b. Nonhost angiosperm volatiles and verbenone disrupt response of western pine beetle, *Dendroctonus brevicomis* (Coleoptera: Scolytidae), to attractant-baited traps. Journal of Economic Entomology, 98, 2041-2048.
13. FETTIG, C. J. & MUNSON, A. S. 2020. Efficacy of verbenone and a blend of verbenone and nonhost volatiles for protecting lodgepole pine from mountain pine beetle (Coleoptera: Curculionidae). Agricultural and Forest Entomology, 22, 373-378.
14. FETTIG, C. J., MUNSON, A. S., REINKE, M. & MAFRA-NETO, A. 2015. A novel semiochemical tool for protecting *Pinus contorta* from mortality attributed to *Dendroctonus ponderosae* (Coleoptera: Curculionidae). Journal of Economic Entomology, 108, 173-182.
15. GILLETTE, N. E., KEGLEY, S. J., COSTELLO, S. L., MORI, S. R., WEBSTER, J. N., MEHMEL, C. J. & WOOD, D. L. 2014. Efficacy of verbenone and green leaf volatiles for protecting whitebark

and limber pines from attack by mountain pine beetle (Coleoptera: Curculionidae: Scolytinae). Environmental entomology, 43, 1019-1026.
16. GILLETTE, N. E., MEHMEL, C. J., MORI, S. R., WEBSTER, J. N., WOOD, D. L., ERBILGIN, N. & OWEN, D. R. 2012. The push-pull tactic for mitigation of mountain pine beetle (Coleoptera: Curculionidae) damage in lodgepole and whitebark pines. Environmental Entomology, 41, 1575-1586.
17. GILLETTE, N. E., MEHMEL, C. J., WEBSTER, J. N., MORI, S. R., ERBILGIN, N., WOOD, D. L. & STEIN, J. D. 2009. Aerially applied methylcyclohexenone-releasing flakes protect *Pseudotsuga menziesii* stands from attack by *Dendroctonus pseudotsugae*. Forest Ecology and Management, 257, 1231-1236.
18. GRAVES, A. D., HOLSTEN, E. H., ASCERNO, M. E., ZOGAS, K. P., HARD, J. S., HUBER, D. P. W., BLANCHETTE, R. A. & SEYBOLD, S. J. 2008. Protection of spruce from colonization by the bark beetle, *Ips perturbatus*, in Alaska. Forest Ecology and Management, 256, 1825-1839.
19. HANSEN, E. M., MUNSON, A. S., WAKARCHUK, D., BLACKFORD, D. C., GRAVES, A. D., STEPHENS, S. S. & MOAN, J. E. 2019. Advances in semiochemical repellents to mitigate host mortality from the spruce beetle (Coleoptera: Curculionidae). Journal of Economic Entomology, 112, 2253-2261.
20. HAYES, J. L., STROM, B. L., ROTON, L. M. & INGRAM, L. L. 1994. Repellent properties of the host compound 4-allylanisole to the southern pine beetle. Journal of Chemical Ecology, 20, 1595-1615.
21. HUNT, D. & BORDEN, J. 1988. Response of mountain pine beetle, *Dendroctonus ponderosae* Hopkins, and pine engraver, *Ips pini* (SAY), to ipsdienol in southwestern British Columbia. Journal of Chemical Ecology, 14, 277-293.
22. JIROŠOVÁ, A., KALINOVÁ, B., MODLINGER, R., JAKUŠ, R., UNELIUS, C. R., BLAŽENEC, M. & SCHLYTER, F. 2022. Anti-attractant activity of (+)-trans-4-thujanol for Eurasian spruce bark beetle *Ips typographus*: Novel potency for females. Pest Management Science, 78, 1992-1999.
23. KOSTYK, B. C., BORDEN, J. H. & GRIES, G. 1993. Photoisomerization of antiaggregation pheromone verbenone: Biological and practical implications with respect to the mountain pine beetle,*Dendroctonus ponderosae* Hopkins (Coleoptera: Scolytidae). Journal of Chemical Ecology, 19, 1749-1759.
24. LINDGREN, B. S. & MILLER, D. R. 2002. Effect of verbenone on five species of bark beetles (Coleoptera: Scolytidae) in lodgepole pine forests. Environmental Entomology, 31, 759-765.
25. LINDMARK, M., WALLIN, E. A. & JONSSON, B.-G. 2022. Protecting forest edges using trap logs–Limited effects of associated push-pull strategies targeting *Ips typographus*. Forest Ecology and Management, 505, 119886.
26. MILLER, D., GRIES, G. & BORDEN, J. 1990. E-myrcenol: a new pheromone for the pine engraver, *Ips pini* (Say) (Coleoptera: Scolytidae). The Canadian Entomologist, 122, 401-406.
27. MILLER, D. R. 2000. Vertical displacement of *Ips latidens* and *Ips pini* (Coleoptera: Scolytidae) by semiochemical interruption. The Canadian Entomologist, 132, 789-797.
28. MILLER, D. R. & ALLISON, J. D. 2011. Variation in enantiospecific attraction of *Ips avulsus* (Coleoptera: Curculionidae) to the pheromone ipsdienol in Georgia. Journal of Economic Entomology, 104, 895-900.
29. MILLER, D. R. & BORDEN, J. H. 2003. Responses of *Ips pini* (Say), *Pityogenes knechteli* Swaine and associated beetles (Coleoptera) to host monoterpenes in stands of Lodgepole Pine. Journal of Entomological Science, 38, 602-611.

30. MILLER, D. R., BORDEN, J. H. & LINDGREN, B. S. 1995. Verbenone: Dose-dependent interruption of pheromone-based attraction of three sympatric species of pine bark beetles (Coleoptera: Scolytidae). Environmental Entomology, 24, 692-696.
31. MUNRO, H. L., GANDHI, K. J. K., BARNES, B. F., MONTES, C. R., NOWAK, J. T., SHEPHERD, W. P., VILLARI, C. & SULLIVAN, B. T. 2020. Electrophysiological and behavioral responses *Dendroctonus frontalis* and *D. terebrans* (Coleoptera: Curculionidae) to resin odors of host pines (*Pinus* spp.). Chemoecology, 30, 215-231.
32. NIÑO-DOMÍNGUEZ, A., SULLIVAN, B. T., LÓPEZ-URBINA, J. H. & MACÍAS-SÁMANO, J. E. 2016. Responses by *Dendroctonus frontalis* and *Dendroctonus mesoamericanus* (Coleoptera: Curculionidae) to semiochemical lures in Chiapas, Mexico: possible roles of pheromones during joint host attacks. Journal of Economic Entomology, 109, 724-731.
33. PAINE, T. D., MILLAR, J. G., HANLON, C. C. & HWANG, J. S. 1999. Identification of semiochemicals associated with Jeffrey Pine Beetle, *Dendroctonus jeffreyi*. Journal of Chemical Ecology, 25, 433-453.
34. PURESWARAN, D. S. & BORDEN, J. H. 2004. New repellent semiochemicals for three species of *Dendroctonus* (Coleoptera: Scolytidae). CHEMOECOLOGY, 14, 67-75.
35. PURESWARAN, D. S., GRIES, R., BORDEN, J. H. & PIERCE, J. H. D. 2000. Dynamics of pheromone production and communication in the mountain pine beetle, *Dendroctonus ponderosae* Hopkins, and the pine engraver, *Ips pini* (Say) (Coleoptera: Scolytidae). Chemoecology, 10, 153-168.
36. RAPPAPORT, N. G., OWEN, D. R. & STEIN, J. D. 2001. Interruption of Semiochemical-mediated attraction of *Dendroctonus valens* (Coleoptera: Scolytidae) and selected nontarget insects by verbenone. Environmental Entomology, 30, 837-841.
37. SAVOIE, A., BORDEN, J. H., PIERCE, H. D., GRIES, R. & GRIES, G. 1998. Aggregation Pheromone of *Pityogenes knechteli* and semiochemical-based interactions with three other bark beetles. Journal of Chemical Ecology, 24, 321-337.
38. STROM, B., SMITH, S. & BROWNIE, C. 2013. Attractant and disruptant semiochemicals for *Dendroctonus jeffreyi* (Coleoptera: Curculionidae: Scolytinae). Environmental Entomology, 42, 323-332.
39. STROM, B. L., ROTON, L. M., GOYER, R. A. & MEEKER, J. R. 1999. Visual and semiochemical disruption of host finding in the southern pine beetle. Ecological Applications, 9, 1028-1038.
40. SULLIVAN, B. T. 2005. Electrophysiological and behavioral responses of *Dendroctonus frontalis* (Coleoptera: Curculionidae) to volatiles isolated from conspecifics. Journal of Economic Entomology, 98, 2067-2078.
41. SULLIVAN, B. T. & BROWNIE, C. 2022. The role of wind and semiochemicals in mediating switching behavior in the Southern Pine Beetle (Coleoptera: Curculionidae: Scolytinae). Environmental Entomology, 51, 340-350.
42. SULLIVAN, B. T., DALUSKY, M. J., MORI, K. & BROWNIE, C. 2011. Variable responses by southern pine beetle, *Dendroctonus frontalis* Zimmermann, to the pheromone component *endo*-brevicomin: influence of enantiomeric composition, release rate, and proximity to infestations. Journal of chemical ecology, 37, 403-411.
43. SULLIVAN, B. T., DALUSKY, M. J., WAKARCHUK, D. & BERISFORD, C. W. 2007. Field evaluations of potential aggregation inhibitors for the southern pine beetle, *Dendroctonus frontalis* (Coleoptera: Curculionidae). Journal of Entomological Science, 42, 139-149.
44. UNELIUS, C. R., SCHIEBE, C., BOHMAN, B., ANDERSSON, M. N. & SCHLYTER, F. 2014. Non-host volatile blend optimization for forest protection against the european spruce bark beetle, *Ips typographus*. PLoS ONE, 9.

45. WERNER, R. A. & HOLSTEN, E. H. 2002. Use of semiochemicals of second bark beetles to disrupt spruce beetle attraction and survival in Alaska. USDA FOREST SERVICE PACIFIC NORTHWEST RESEARCH STATION RESEARCH PAPER PNW-RP.
46. WILSON, I., BORDEN, J., GRIES, R. & GRIES, G. 1996. Green leaf volatiles as antiaggregants for the mountain pine beetle, *Dendroctonus ponderosae* Hopkins (Coleoptera: Scolytidae). Journal of Chemical Ecology, 22, 1861-1875.
47. ZHANG, L., CLARKE, S. R. & SUN, J. 2009a. Electrophysiological and behavioral responses of *Dendroctonus valens* (Coleoptera: Curculionidae: Scolytinae) to four bark beetle pheromones. Environmental Entomology, 38, 472-477.
48. ZHANG, L. W., GILLETTE, N. E. & SUN, J. H. 2007a. Electrophysiological and behavioral responses of *Dendroctonus valens* to non-host volatiles. Annals of Forest Science, 64, 267-273.
49. ZHANG, Q.-H., SCHLYTER, F., CHEN, G. & WANG, Y. 2007b. Electrophysiological and behavioral responses of *Ips subelongatus* to semiochemicals from its hosts, non-hosts, and conspecifics in China. Journal of Chemical Ecology, 33, 391-404.
50. ZHANG, Q.H., SONG, L.W., MA, J.H., HAN, F.Z. & SUN, J.H. 2009b. Aggregation pheromone of a newly described spruce bark beetle, *Ips shangrila* Cognato and Sun, from China. Chemoecology, 19, 203-210.
51. ZHANG, Q. H., LIU, G. T., SCHLYTER, F., BIRGERSSON, G., ANDERSON, P. & VALEUR, P. 2001. Olfactory responses of *Ips duplicatus* from inner Mongolia, China to nonhost leaf and bark volatiles. Journal Of Chemical Ecology, 27, 995-1009.
52. ZHANG, Q. H. & SCHLYTER, F. 2003. Redundancy, synergism, and active inhibitory range of non-host volatiles in reducing pheromone attraction in European spruce bark beetle *Ips typographus*. Oikos, 101, 299-310.
