# Supplementary material for: How Effective Are Push–Pull Semiochemicals as Deterrents for Bark Beetles? A Global Meta-Analysis of Thirty Years of Research
Source: Insects. 2023 Oct 13;14(10):812. doi: 10.3390/insects14100812 (PMC10607257; doi:10.3390/insects14100812)
Supplement: Supplementary file 1 [file insects-14-00812-s001.zip › S3_Table.pdf]

Table S3. Total number of manuscripts describing the effect of push-pull semiochemical treatment on attack and attraction of *Dendroctonus* and *Ips* species

Number of manuscripts*		
**Variable**	*Dendroctonus*	*Ips*
Tree killed	4	0
Tree attack	10	2
Attraction to lure-male	11	3
Attraction to lure-female	12	3
Attraction to lure-both sexes	25	17

*Total number of manuscripts is 52, but some manuscripts describe more than one type of measure.
